# Role of Dietary Components in Modulating Hypertension

**DOI:** 10.4172/2155-9880.1000433

**Published:** 2016-04-24

**Authors:** Andrew Feyh, Lucas Bracero, Hari Vishal Lakhani, Prasanna Santhanam, Joseph I Shapiro, Zeid Khitan, Komal Sodhi

**Affiliations:** 1 Department of Medicine, Joan C. Edwards School of Medicine, Marshall University, USA; 2 Department of Biological Science, Marshall University, USA; 3 Department of Surgery and Pharmacology, Joan C. Edwards School of Medicine, Marshall University, USA

**Keywords:** Hypertension, Diet, Modulation, Blood pressure, Serum potassium, *Capsicum* genus

## Abstract

Hypertension is a major health issue, particularly in medically underserved populations that may suffer from poor health literacy, poverty, and limited access to healthcare resources. Management of the disease reduces the risk of adverse outcomes, such as cardiovascular or cerebrovascular events, vision impairment due to retinal damage, and renal failure. In addition to pharmacological therapy, lifestyle modifications such as diet and exercise are effective in managing hypertension. Current diet guidelines include the DASH diet, a low-fat and low-sodium diet that encourages high consumption of fruits and vegetables. While the diet is effective in controlling hypertension, adherence to the diet is poor and there are few applicable dietary alternatives, which is an issue that can arise from poor health literacy in at-risk populations. The purpose of this review is to outline the effect of specific dietary components, both positive and negative, when formulating a dietary approach to hypertension management that ultimately aims to improve patient adherence to the treatment, and achieve better control of hypertension.

## Introduction

Hypertension is estimated by the Centers for Disease Control and Prevention to affect approximately 30% of adults in the United States [1]. It is a significant factor in the pathogenesis of a number of diseases, including obesity, cardiovascular disease, and stroke [2–4]. Hypertension itself can be caused by unhealthy lifestyle habits like alcoholism, drug addiction, smoking, high stress, or obesity, and certain non-modifiable attributes like age, gender, hereditary and genetic constitution, and racial or ethnic disparities.

Hypertension to an extent correlates to the prevailing socioeconomic and geographical characteristics of a region, as well as to individual behavioral factors, and can pose a significant public health concern in populations defined by economic hardship, poverty, reduced health care access, low health literacy, and lack of resources due to geographic isolation [5–7]. For example, in the United States, the prevalence of obesity is greatest in Appalachia, with West Virginia leading the national rankings with 35.1% of adults with obesity and 41% with hypertension [8].

Treatment of hypertension centers on managing blood pressure within a target range while trying to minimize drug-associated side effects. Treatment protocol for hypertension is evolving, with guidelines set forth by the JNC 7 in 2004, and again modified in the JNC 8 in 2014. The JNC 7 defined blood pressure control as <140/90 mmHg, or <130/80 mmHg in those with kidney disease or diabetes, whereas the JNC 8 suggested new standards in patients ≥ 60 years of age without kidney disease or diabetes to <150/90 mmHg [9]. However, the recent SPRINT trial has taken a more aggressive approach to blood pressure control, defining standard control as systolic blood pressure (SBP) <140 mmHg, intensive control as SBP <120 mmHg, and primary composite outcome as a cardiovascular or cerebrovascular event, heart failure, or death. This trial demonstrated for intensive treatment a hazard ratio of 0.75 with a 95% confidence interval (CI) of 0.64 to 0.89 (p<0.001) for primary composite outcomes, and an all-cause mortality hazard ratio of 0.73 (95% CI: 0.6 to 0.9, p=0.003). However, intensive therapy was associated with an increased risk of adverse side effects, such as hypotension, syncope, and electrolyte disturbances [10]. To achieve blood pressure control, a wide array of pharmacological interventions is employed. Treatment with antihypertensive drugs can reduce the risk of stroke by over 40% and the risk of heart failure by approximately 50% [11,12].

Among the lifestyle and diet modifications that are recommended, one that is recommended to all persons with hypertension, regardless of pharmacological intervention, is the DASH (Dietary Approaches to Stop Hypertension) diet, a diet that advocates the consumption of fiber and potassium via fruits and vegetables, reduction in total and saturated fat via a reduction in meat and animal products, and intake of adequate protein via lean meat and low-fat dairy products instead of high fat or processed meat [13]. This diet has been demonstrated to be effective in managing blood pressure, with the original DASH feeding trial demonstrating a decrease in SBP of 11.4 mmHg and a decrease in diastolic blood pressure (DBP) of 5.5 mmHg compared to control subjects [14]. However, a meta-analysis by Kwan et al. has demonstrated significant challenges in maintaining adherence to the protocol [14]. These findings are echoed in a study by Lin et al. that showed that only 52% of obese adults in 2007 were advised by their physician on healthy eating, and those who received no counseling were less likely to adopt healthy eating practices (33% of uncounseled versus 78% of counseled patients). Counseling by physicians and lifestyle coaching by other professionals improved adherence to the DASH diet, indicating the important role that the physician and ancillary staff plays in promoting dietary changes as a component of hypertension therapy [15].

## Antihypertensive Dietary Components

### Fruits and vegetables

Certain diets rich in fruits and vegetables, such as the vegetarian diet and the Mediterranean Diet, have demonstrated efficacy in controlling blood pressure [16]. The mechanisms by which fruits and vegetables are able to help manage blood pressure vary according to their potassium content, flavonoid and polyphenol content, and fiber content. Serum potassium levels have been shown to be inversely associated with blood pressure, with antihypertensive dietary interventions encouraging decreases in sodium consumption and increases in potassium consumption in order to improve the sodium-potassium ratio in the diet [17–19].

Pomegranate juice is rich in phytochemicals and polyphenols, which have antioxidant and anti-inflammatory functions that have been reported to promote cardiovascular health [20,21]. Pomegranate juice may be able to prevent the development of high blood pressure due to angiotensin II in diabetes via inhibition of angiotensin converting enzyme (ACE) [22]. Experimental data as well as clinical trials have shown that consumption of pomegranate juice has antihypertensive effects [20, 21,23]. In a study of 13 hypertensive men aged 39–68, 150 mL/day of natural pomegranate juice resulted in a significant reduction of SBP (7%, from 125.38 ± 11.80 to 116.15 ± 7.94, p=0.013) and DBP (6%, from 82.69 ± 5.25 to 78.08 ± 3.25, P <0.010) [21]. In a study by Mohan et al. daily administration of 100 mg/kg and 300 mg/kg of pomegranate juice extract in diabetic Wistar rats for 4 weeks was found to reduce the main arterial blood pressure and vascular reactivity changes to various catecholamines; reversal of biochemical changes induced by diabetes and angiotensin II were also observed [23]. Due to the antihypertensive effects and promotion of cardiovascular health, pomegranate juice may be considered as a dietary form of management for hypertension.

Citrus fruits, such as oranges and grapefruits, have also demonstrated antihypertensive properties. A single-blind randomized crossover study of 22 healthy patients aged 18–59 showed that after drinking 500 ml/day of commercial citrus sinensis juice, both DBP and SBP decreased (−5.13%, P=0.03 and −5.91%, P=0.0003, respectively), while natural orange juice did not show significant effects [24]. Citrus paradisi juice, from grapefruit, has many beneficial properties and is a good source of vitamin C, pectin fiber, and antioxidants. Grapefruit juice has been shown to decrease both SBP and DBP in both normotensive and hypertensive human subjects [25]. Extract from grapefruit peels is high in polyphenols, which act as strong inhibitors of α-glucosidase and can be used in the management of hypertension [26]. A study indicated that when compared, grapefruit juice produced a greater decrease in mean arterial pressure than orange juice [25]. These studies indicate that fruits and vegetables can help manage blood pressure through a wide variety of mechanisms, and should play a major role in an antihypertensive dietary intervention.

Garlic is a vegetable that has been investigated for its cardiovascular benefits. In addition to its antioxidant properties, the antihypertensive effects of garlic have been linked to its allicin content, a sulfur compound that can inhibit angiotensin II and promote vasodilation [27]. A meta-analysis of 10 studies on garlic and hypertension demonstrated a decrease in SBP of 8.4 ± 2.8 mmHg (p<0.001) and a decrease in DBP of 7.3 ± 2.5 mmHg (p<0.001) among hypertensive patients compared to placebo, though the form of garlic varied from powder to oil to extract [27]. Another study by Ried, et al. evaluated the effect that garlic extract supplementation had on the blood pressure of 79 hypertension patients. The study showed a reduction in mean SBP of 11.8 ± 5.4 mmHg (p=0.006) in patients taking two daily capsules of garlic extract (1.2 mg S-allylcysteine) for 12 weeks compared to placebo, a therapy regimen that was well-tolerated by the cohort [28]. A meta-analysis by Wang et al. showed that garlic supplementation in hypertensive patients resulted in a reduction in SBP of −4.4 mmHg (95% CI: −7.37 to −1.42; p=0.004), and a reduction in DBP of −2.68 mmHg (95% CI: −4.93 to −0.42; p=0.02) [29]. The studies within the meta-analysis used garlic powder, garlic extract, and garlic oil as garlic supplements [29]. Additionally, since it is primarily used to enhance the flavor of food, it can reduce the desire to use salt as a seasoning, presenting the patient with a palatable option for reducing salt intake. In summary, garlic, due to multifactorial effects, may be used to lower blood pressure.

### Dairy products

Dairy products are major sources of protein and nutrition within vegetarian diets and the Mediterranean Diet, both of which have been shown to be effective in controlling blood pressure [16]. The mechanisms by which dairy products help to manage blood pressure are not entirely clear, but it is suspected that they correlate with the magnesium and potassium contents of these food sources. Additionally, bioactive peptides called lactotripeptides within dairy products can contribute to the antihypertensive effects associated with their consumption [16].

In a study by Drouin-Chartier et al., 76 weight-stable adults with mild-to-moderate hypertension consumed three daily servings of dairy products or a control that was equivalent in macronutrient and sodium content during four-week treatment periods. The study demonstrated an association between dairy consumption and a significant decrease in ambulatory SBP in men (−2 mm Hg, p=0.05), though no significant change in ambulatory DBP was observed (−1 mm Hg, p=0.37). The observed effect on SBP was not significant in women. However, endothelial function, as measured by the reactive hyperemia index (RHI) significantly improved in both men and women consuming dairy compared to controls when adjusted for energy intake (+0.09, p=0.04) [16]. In the Rotterdam study, 2245 normotensive adults over the age of 55 were assessed at baseline and after a 2-year follow-up and 6-year follow-up. This study demonstrated an inverse correlation between low-fat dairy consumption and the incidence of hypertension seen at 6-year follow-up: the hazard ratios (HR) and 95% confidence interval (CI) across quartiles of increasing dairy consumption were 1.00; 0.89 (0.71 to 1.15); 0.84 (0.66 to 1.08); and 0.76 (0.59 to 0.97) in overweight subjects (p=0.03 for trend) [30]. The greatest effect was seen in consumption of low-fat milk and dairy products, and little risk reduction was seen in consumption of high-fat dairy products and cheese [30]. These results suggest that low-fat dairy products may be useful in controlling hypertension, particularly among men and the overweight, and should be considered within a dietary intervention.

### Spices

Spices, including chili peppers, cinnamon, black pepper, and turmeric, can also have antihypertensive effects. In addition to their flavor-enhancing qualities, these spices have been used for centuries worldwide as herbal medicines within the practice of traditional medicine, and have become the subjects of increased focus within the Western medical paradigm as possible adjunct therapies due to their wide therapeutic indices and minimal side effect profiles.

Capsaicin is the active component in red peppers, yellow peppers, paprika peppers, and other plants in the *Capsicum* genus, and is a natural irritant to humans. The effects of capsaicin on blood pressure have been shown to vary depending on the amount ingested. Studies have indicated that ingestion of large quantities of capsaicin-rich peppers can induce a hypertensive crisis. The proposed explanation for this reaction is via a decreased release of calcitonin gene-related peptide (CGRP) from myocardial C fibers that are sensitive to capsaicin [31,32]. While research has shown that ingestion of large quantities of capsaicin can induce a hypertensive crisis, moderate capsaicin consumption can have antihypertensive effects. Several peppers from the *Capsicum* genus, including bell peppers and paprika, exert a strong inhibitory effect on Angiotensin Converting Enzyme (ACE). This research suggests that peppers of the *Capsicum* genus may be useful as dietary cofactors in hypertension management, and especially in ACE-dependent hypertension. A study performed by Ranilla et al. demonstrated that the ACE inhibitory activity significantly correlates with the total phenolic contents (r=0.61, p<0.05) but not with the antioxidant activity, with paprika and red peppers showing the highest ACE inhibition (92% and 84% respectively) [33]. A similar study by Kwon et al. reported that yellow pepper extracts had the highest ACE inhibitory activity (84.1%) while red pepper extract ACE inhibitory activity was 76.5%, but found no correlation between the total phenolic content and antioxidant activity [34].

Cinnamon has been reported to reduce blood sugar in patients with diabetes, and studies have shown that agents that enhance insulin sensitivity or reduce circulating insulin levels also lower blood pressure. Cinnamon extracts have been shown in several studies to have antihypertensive and vasorelaxant effects in rats. In a study by Nyadjeu et al. acute intravenous administration of the MECZ (5, 10, and 20 mg/kg) showed long-lasting decrease in blood pressure (12.5%, 26.6%, and 30.6% respectively) in L-NAME-induced hypertensive rats [35]. In another study by Nyadjeu et al. intravenous administration of MECZ induced a significant (p<0.001) decrease in mean arterial blood pressure in Wistar rates that has induced arterial hypertension from 20 mg/kg L-NAME injections [36]. The antihypertensive effects of Cinnamon extracts may be a result of its ability to increase the production of endogenous nitrous oxide (NO) or to regulate dyslipidemia [36]. The addition of cinnamon to both high sucrose-containing diets, which have known correlations to insulin resistance and elevated blood pressure, and non-sucrose containing diets of rats with spontaneous hypertension, has been shown to reduce SBP in a dose-dependent manner. Preuss et al. found that the addition of 8% weight/weight of cinnamon to the diets of rats eating sucrose lowered the SBP to essentially that of the rate on starch only diets [37].

Black pepper is one of the most ubiquitous spices used in cuisine worldwide. The essential oils within black pepper have known antioxidant activity as well as the ability to inhibit ACE [38]. Black pepper contains piperine, which is the chemical compound responsible for its pungency. Piperine supplementation has been demonstrated to normalize blood pressure and improve glucose tolerance, as well as have positive cardiac and liver effects in rats on a high fat, high carbohydrate diet that developed hypertension, elevated oxidative stress and cardiac changes. Diwan et al. showed that piperine normalized SBP after 8 weeks of treatment in rats fed a high carbohydrate high fat diet (p <0.05) [39]. Piperine has also been shown to partially prevent the increase in blood pressure in L-NAME-induced hypertension, most likely via the blockade of voltage-dependent calcium channels [40].

Curcumin, the active ingredient in turmeric, has many potential beneficial effects on blood pressure. It has been shown to inhibit hypertension-induced increase in myocardial cell diameter as well as significant inhibition in perivascular fibrosis in rat models, and could therefore be a therapeutic agent for heart failure, which can arise from chronic uncontrolled hypertension [41]. Curcumin is also able to partially prevent the increase in blood pressure caused by L-NAME, NO-deficient hypertension, as well as prevent elastin decrease in the aorta, which is important for maintaining vessel elasticity and preventing arteriosclerosis [42]. In a study by Hlavackova et al. administration of L-NAME increased the blood pressure in Wistar rats to 157.4 mmHg, with further administration increasing this value more; curcumin administration was able to stop the increase in blood pressure caused by the L-NAME administration, while causing a decrease of 28.9 mmHg throughout the experiment [42]. Nakmareong et al. determined that the effect of curcumin on SBP during L-NAME administration for 3 weeks, showing a significant decrease in SBP (p<0.05) when compared with the L-NAME control group [43]. Curcumin’s ability to prevent development of vascular dysfunction in NO-deficient hypertension is thought to be associated with its antioxidant properties [43].

In summary, when formulating an antihypertensive diet, certain spices can be helpful in the reduction and maintenance of blood pressure. Like garlic, these spices enhance flavor for greater palatability, and may help in lowering salt intake.

### Green tea

Green tea is a commonly consumed beverage known for its widespread health benefits. Of particular interest is a group of polyphenols within green tea known as catechins, of which the key one is epigallocatechin gallate (EGCG). EGCG stimulates nitric oxide synthesis in endothelial cells to promote vasodilation and reduces synthesis of endothelin-1, a vasoconstrictor, to improve overall vascular function [44,45]. Additionally, EGCG might have an inhibitory effect on the action of renin. By inhibiting this system at the rate-limiting step of renin action, EGCG is able to effectively inhibit fluid retention and vasoconstriction [46]. Additionally, by upregulating HO-1, an enzyme also upregulated in response to stress and inflammation, EGCG can exert a greater anti-inflammatory effect to protect against endothelial damage and dysfunction [45].

In a longitudinal population study by Tong et al. conducted on 1109 Chinese adults, green tea consumption was demonstrated to inversely correlate with changes in blood pressure over a five-year period in a dose-dependent manner [47]. Among those who consumed the most green tea (>10 g/day), DBP changes were minimized in nonsmoking subjects (>10 g/day: −7.6 mmHg (95% CI: −11.96 to −3.24) versus <10 g/day: −3.43 mmHg (95%CI: −6.19 to −0.68), p<0.01 for trend), whereas both SBP and DBP changes were minimized in nonobese nonsmoking subjects [47]. However, subjects who smoked had a muted response to green tea consumption [47]. Green tea consumption, or supplementation with green tea flavonoids, could serve as an alternative to sugar-sweetened beverages within the framework of a diet to control hypertension.

### Omega-3 fatty acids

Omega-3 fatty acids (n−3FAs) have been extensively studied for their cardiovascular health benefits. The major types of omega-3 fatty acids include eicosapentaenoic acid (EPA) and docosohexaenoic acid (DHA) found primarily in seafood and fish oil, and alpha-linolenic acid (ALA) found primarily in plant sources such as flaxseed and walnuts [48]. Omega-3 fatty acids are able to exert their antihypertensive effects by regulating inflammatory signaling through modulation of the expression of cytokines and prostaglandins with vasoactive properties. N-3FAs accomplish this is by interaction with the cyclooxygenase-1 (COX-1) enzyme to moderate prostaglandin synthesis [49]. N-3FAs are also believed to regulate the levels of oxylipins, which are vasoactive compounds produced by soluble epoxide hydrolase derived from fatty acids. The oxylipins derived from arachidonic acid or linoleic acid can have vasoconstrictive and inflammatory effects, but n−3FAs can inhibit the activity of soluble epoxide hydrolase, thereby reducing the production of such oxylipins [50].

In the INTERMAP study, a population study of 4680 adults aged 40–59 from China, Japan, the United Kingdom, and the United States, the dietary n−3FA intake was studied to determine its effect on blood pressure. Participants were observed on two consecutive days at baseline, then two consecutive days three weeks later. The results showed an inverse correlation among all participants between dietary n−3FA intake and both SBP and DBP after three weeks (>1.9 g/day n−3FA: −0.55 mmHg SBP and −0.57 mmHg DBP), with ALA, DHA, and EPA all demonstrating antihypertensive effects [51]. In a longitudinal population study by Xun et al. of 4508 American adults aged 18–30 without hypertension at baseline, the incidence of hypertension was monitored over a 20-year course. This study demonstrated that among those in the highest quartile of n−3FA consumption (>0.37 g/day), the incidence of hypertension was significantly lower at follow-up when compared to those in the lowest quartile (HR=0.65 (95% CI: 0.53–0.79), p<0.01 for the trend) [52]. These studies suggest that n−3FAs can result in modest SBP and DBP reduction when supplemented or incorporated into an antihypertensive intervention diet.

### Trace elements

Several trace elements, such as copper and zinc, play an important role in maintaining blood pressure. The exact method is not clear but abnormal copper and zinc metabolism have been implicated as risk factors for development of hypertension and vascular disease [53–55].

Serum calcium levels have been shown to be associated with cardiovascular disease (CVD) [56], but the relation between calcium and hypertension is not clear. Some studies, but not all, have shown that increased intake of dietary calcium, and magnesium, reduces blood pressure in patients with hypertension [57]. However, these results have been seen only in dietary intake and not through supplementation [58].

Magnesium affects blood pressure by modulating vascular tone and reactivity. Hypomagnesemia is associated with diabetes and metabolic syndrome, with magnesium playing a major role is regulation of blood pressure [59]. Magnesium affects blood pressure by modulating vascular tone and reactivity. As with calcium, the data is not consistent, but there appears to be an inverse relationship with dietary, not supplemental, magnesium intake and blood pressure [60]. While experimental data supports that low magnesium levels leads to hypertension, clinical observation and trials have provided some conflicting results [61]. While clinical trials have shown conflicting results, there are studies that have demonstrated a significant association between hypomagnesemia and prehypertension and hypertension. In a study by Guerrero-Romero et al. of 3954 healthy Mexican children, hypomagnesemia was observed in 59 children with prehypertension (27.3%) and 52 (45.6%) with hypertension in the 6–10 age group and 115 children with prehypertension (36%) and 109 (49.6%) with hypertension in the 11–15 age group. Regression analysis from the Guerrero-Romero study showed that hypomagnesemia was associated with both prehypertension (6–10 years: p<0.0005; 11–15 years: p=0.018) and hypertension (6–10 years: p <0.0005; 11–15 years: p=0.0002) [62].

## Prohypertensive Dietary Components

### Fructose

Fructose is a sugar monomer that can exist in its free monomer form, bound to glucose as a component of the sucrose dimer, and in high levels in sweeteners such as high-fructose corn syrup (HFCS). Common dietary sources of fructose include fruits and sugar-sweetened beverages, as well as sources of sucrose, which is commonly processed into table sugar from sugarcane. It has been a source of dietary controversy, with excess consumption being linked to metabolic disorders [63]. In the laboratory setting, fructose has been demonstrated to induce hypertension and metabolic syndrome in rat models [64]. One of the mechanisms by which fructose may be implicated in hypertension is its contribution to elevated uric acid levels. Metabolism of fructose is governed by an enzyme called fructokinase, also known as ketohexokinase (KHK). Phosphorylation of fructose is achieved by transferring a phosphate group from ATP, but since KHK lacks a negative feedback mechanism, ATP is quickly depleted, with the resulting dephosphorylated purines degraded by AMP deaminase. This degradation results in increased uric acid levels [63]. Hyperuricemia has been demonstrated in multiple studies to correlate with hypertension [65–69], and so by this mechanism, it would appear that excess fructose consumption would lead to hypertension.

Demonstrating this link between fructose consumption and hypertension within human populations, however, has been controversial. Some studies have been able to demonstrate a link. In a cross-sectional study by Jalal et al. of 4528 adults, 1754 of whom were prehypertensive or had Stage 1 or Stage 2 hypertension, fructose consumption was determined by dietary survey and its relation to blood pressure was assessed. The median fructose consumption in this study was 74 g/day, with consumption of at least 74 g/day demonstrating an increased risk of elevated blood pressure (26% increase for ≥ 135/85 mmHg, 30% increase for ≥ 140/90 mmHg, and 77% increase for ≥ 160/100 mmHg) [64]. In an INTERMAP study by Brown et al. 2696 adults aged 40–59 from the United States and United Kingdom, increased fructose consumption via sugar-sweetened beverages was associated with a significantly increased risk of hypertension in a dose dependent manner, with each 355 ml/day increase in sugar-sweetened beverage intake corresponding to an increase in SBP of +1.1 mmHg (p<0.001) and DBP of +0.4 mmHg (p<0.05) when adjusted for subject weight and height. Furthermore, fructose intake two standard deviations from the mean was associated with an increase in SBP of +2.5 mmHg (p=0.002) and an increase in DBP of 1.7 mmHg (p=0.002) when adjusted for subject weight and height [70].

In contrast, other studies have not demonstrated a link between fructose consumption and hypertension. In one study of 267 adults, subjects were fed sugar-sweetened milk containing fructose, glucose, sucrose, or HFCS, for 10 weeks. This study found no significant changes in blood pressure or uric acid levels between groups. However, this study administered fructose in levels associated with the 50th percentile population consumption level (49 ± 1.0 g/day as determined in a study by Marriott, Cole, and Lee), and did not test the extremes of fructose consumption that could be occurring within the standard American diet [71,72]. Another study analyzed data from three major longitudinal studies of over 200,000 people in total with each spanning from 14–20 years: the Nurses’ Health Study 1 (NHS1), Nurses’ Health Study 2 (NHS2), and the Health Professionals’ Follow-Up Study (HPFS). Analysis of these studies did not demonstrate a significant link between fructose consumption and the incidence of hypertension, with an NHS1 relative risk of 1.02 (95% CI: 0.99–1.06); an NHS2 relative risk of 1.03 (95% CI: 0.98–1.08); and an HPFS relative risk of 0.99 (95% CI: 0.93–1.05) [73]. Though the link between fructose consumption and hypertension may still be controversial, the mechanisms by which fructose could possibly contribute to hypertension are plausible enough that controlling the consumption of sugar-sweetened beverages and added sugar within the framework of an antihypertensive diet could be justified.

### Sodium

Sodium has long been implicated in the development and progression of hypertension. However, the mechanisms by which it may contribute to hypertension are not fully clear. Sodium does play a major role in fluid balance, and dysregulation in this process can contribute to excess fluid retention, resulting in hypertension. Additionally, sodium can also induce the production of vasoconstrictive prostaglandins. This is a result of the action of sodium on renal medullary interstitial cells, promoting NFκB signaling and COX-2 expression and leading to increased levels of PGE2 [74].

Dietary sodium restriction is one of the foundations of antihypertensive diets. In a study of 412 adults that were prehypertensive or mildly hypertensive, reductions in dietary sodium were associated with decreases in SBP, with the greatest decreases associated with the lowest sodium intakes. Compared to a high-sodium control diet, a low-sodium (≤65 mmol/day) DASH diet was associated with a decrease in SBP of 11.5 mmHg in patients with hypertension, and a decrease in SBP of 7.1 mmHg in patients without hypertension [75]. In a meta-analysis of 34 trials encompassing 3230 subjects, the mean reduction of 75 mmol/day of sodium (4.4 g/day) resulted in a mean change in SBP of −4.18 mmHg (95% CI: −5.18 to −3.18) and a mean decrease in DBP of 2.06 mmHg (95% CI: −2.67 to −1.45). The relationship between decreased sodium intake and decreased blood pressure was observed in both hypertensive and normotensive subjects, and was demonstrated regardless of sex or ethnicity [76]. These studies suggest that managing sodium intake is a critical component in managing hypertension, and that an antihypertensive diet should emphasize a reduction in dietary sodium (Table 1).

## Conclusion

The DASH diet is a safe and powerful tool in the management of hypertension, and patient education by the physician and other health professionals can play a major role in encouraging adherence to the diet [14,15]. In counseling the patient, the physician must be educated with regards to effective dietary strategies in order to present a specific and coherent dietary plan, and present the patient with a wide array of palatable options that can help encourage adherence to the intervention. Education of the patient can thereby empower them with the requisite health literacy to manage their blood pressure through lifestyle modification. Particularly within medically underserved populations, who are disproportionately affected by hypertension, improved health literacy and patient self-sufficiency can have a major impact on the management of this disease.

## Figures and Tables

**Table 1:** This table summarizes the dietary components reviewed in this paper and their net effect on hypertension.

Dietary Component	Effect on Hypertension
**Fruits and Vegetables:**	
Pomegranate Juice	↓
Citrus Fruit (e.g. oranges and pomegranate)	↓
Garlic	↓
Dairy Products	↓
Spices:	
Chili Peppers (e.g. red peppers, yellow peppers, paprika)	↓/↑*
Cinnamon	↓
Black Pepper	↓
Turmeric	↓
Green Tea	↓
Omega-3 Fatty Acids	↓
Trace Elements:	
Copper	↓
Zinc	↓
Magnesium	↓
Calcium	↓
Sodium	↑
Fructose	↑

* The effect of chili peppers on hypertension varies based on the consumed amount. Consumed in large quantities, chili peppers can promote hypertension, but when consumed in moderate quantities, they have antihypertensive effects.
